# An Emperor Penguin Population Estimate: The First Global, Synoptic Survey of a Species from Space

**DOI:** 10.1371/journal.pone.0033751

**Published:** 2012-04-13

**Authors:** Peter T. Fretwell, Michelle A. LaRue, Paul Morin, Gerald L. Kooyman, Barbara Wienecke, Norman Ratcliffe, Adrian J. Fox, Andrew H. Fleming, Claire Porter, Phil N. Trathan

**Affiliations:** 1 British Antarctic Survey, Cambridge, United Kingdom; 2 Polar Geospatial Center, University in Minnesota, Minneapolis, Minnesota, United States of America; 3 Scripps Institution of Oceanography, University of California San Diego, La Jolla, California, United States of America; 4 Australian Antarctic Division, Hobart, Tasmania, Australia; Phillip Island Nature Parks, Australia

## Abstract

Our aim was to estimate the population of emperor penguins (*Aptenodytes fosteri*) using a single synoptic survey. We examined the whole continental coastline of Antarctica using a combination of medium resolution and Very High Resolution (VHR) satellite imagery to identify emperor penguin colony locations. Where colonies were identified, VHR imagery was obtained in the 2009 breeding season. The remotely-sensed images were then analysed using a supervised classification method to separate penguins from snow, shadow and guano. Actual counts of penguins from eleven ground truthing sites were used to convert these classified areas into numbers of penguins using a robust regression algorithm.

We found four new colonies and confirmed the location of three previously suspected sites giving a total number of emperor penguin breeding colonies of 46. We estimated the breeding population of emperor penguins at each colony during 2009 and provide a population estimate of ∼238,000 breeding pairs (compared with the last previously published count of 135,000–175,000 pairs). Based on published values of the relationship between breeders and non-breeders, this translates to a total population of ∼595,000 adult birds.

There is a growing consensus in the literature that global and regional emperor penguin populations will be affected by changing climate, a driver thought to be critical to their future survival. However, a complete understanding is severely limited by the lack of detailed knowledge about much of their ecology, and importantly a poor understanding of their total breeding population. To address the second of these issues, our work now provides a comprehensive estimate of the total breeding population that can be used in future population models and will provide a baseline for long-term research.

## Introduction

An accurate assessment of the emperor penguin (*Aptenodytes fosteri*) population is urgently needed as recent research indicates that numbers may decrease significantly in coming decades [1], [2], [3]. These studies have highlighted the susceptibility of emperor penguins to changes in sea ice distribution. Recent recorded changes in sea-ice are substantial [4] and predictions suggest sea ice variation will increase with predicted climate change [5], [6]. The subsequent change in marine food webs [7], and other possible developments linked to climate change such as increased predation [8], increased competition, and an increasing frequency of storm events is likely to impact on their breeding success and colony viability [4], [9], [10]. The loss of one colony has already been attributed to climatic warming and others are thought to be vulnerable [4], especially those in the north of the species' breeding range [2] or those currently experiencing regional climate change [8].

One of the most important parameters of any population assessment model is knowledge of the extant population size and status of the breeding colonies [11], [12]. These parameters are the starting point for any demographic model. For emperor penguins this knowledge is limited; only five colonies are monitored each year, but these colonies are geographically restricted to the Ross Sea area and the East Antarctic coast between longitudes 20°E and 140°E. The regional nature of climate change in Antarctica [5] means that a more extensive knowledge of population and population dynamics is required, particularly in those areas where climate change is most evident. For much of the emperor penguins geographic range we have little or no information on demographic change. The paucity of data regarding population status of emperor penguins is largely due to the logistical difficulties of accessing potential emperor penguin breeding habitat in areas of Antarctica that are not in close proximity to research stations. The last global population estimate of 135,000–175,000 pairs [13], compiled nearly two decades ago, was based on a compendium of previous reports. However, the accuracy and validity of many of the counts used to compile this figure have been questioned [14]. Further, many colonies have not previously been counted, including the ten new locations reported in a recent Landsat survey [15] and the new colonies found in our study. Also, many of the colonies where counts do exist were last counted several decades ago (Table 1), while other counts rely on estimates from late in the breeding season (i.e. after an unknown number of eggs and chicks had already been lost and adults may have already departed from the colony) [16]. These concerns over the lack of a baseline population figure for the species have led to the suggestion that emperor penguins should be re-classified by the IUCN from ‘of least concern’ to ‘data deficient’ [14].

**Table 1:** Emperor penguin population survey 2009 results.

Name	long	lat	image date	area (m^2^)	BE	image notes	PLC	source	notes
Cape Colbeck, Edward VII Peninsula	−157.7	−77.14	13/10/2009	12262	11438	good	6358	[16]	
Rupert Coast	−143.3	−75.38	20/10/2008	1660	1550	good	Uncounted		
Ledda Bay	not found		27/10/2009	0	0	NA	Uncounted		Sea ice gone before image taken
Thuston Glacier, Mt Siple	−125.621	−73.5	17/10/2009	3205	2989	good	2500	[11] chick estimate	Previous count very late in the season
Bear Peninsula	−110.25	−74.35	18/11/2009	10144	9457	good	Uncounted		
Brownson Islands	−103.64	−74.35	18/11/2009	6140	5732	poor	Uncounted		Heavy guano
Noville Peninsula	−98.45	−71.77	17/11/2009	3822	3568	poor	Uncounted		Heavy guano
Smyley	−78.83	−72.3	12/11/2009	6496	6061	good	Uncounted		
Smith	−60.83	−74.37	30/10/2009	4307	4018	good	Uncounted		
Dolleman	−60.43	−70.61	04/10/2009	1737	1620	good	Uncounted		Small part of colony missing in image
Snowhill	−57.44	−64.52	26/10/2009	2321	2164	poor	3885	[19]	
Gould	−47.68	−77.71	14/10/2009	8833	8242	good	7500	[34]	
Luitpold	−33.6	−77077	12/11/2009	6969	6498	good	Uncounted		
Dawson	∼−26.67	∼−76.02	13/10/2009	2784	2597	good	11700	Asplin -unpublished BAS report 1986	
Halley	−27.43	−75.54	27/10/2009	24127	22510	good	14300	Asplin -unpublished BAS report 1987	
Stancomb	−23.09	−74.12	21/10/2009	5849	5455	fair	3000	Asplin –unpublished BAS report 1986	Small amount of smearing
Drescher	−19.34	−72.83	04/10/2009	2469	2305	fair	6600	[35]	No guano, analysis on panchromatic band only
Riiser	−15.11	−72.12	27/10/2009	4304	4013	fair	5900	[35]	High cloud- cover
Atka	−8.13	−70.61	08/09/2009	10355	9657	good	8000	[35]	
Sanae	−1.42	−70	28/10/2009	3423	3193	good	113	[36]	
Astrid	8.31	−69.95	28/11/2009	1467	1368	poor	Uncounted		Late image, colony already dispersed
Lazarev	15.55	−69.75	11/10/2009	881	821	fair	4500	[37]	
Ragnhild	27.15	−69.9	10/10/2009	7362	6870	good	Uncounted		
Gunnerus	34.38	−68.75	31/10/2009	4989	4652	fair	7000	[28]	
Umbeashi	43.01	−68.05	14/10/2009	156	146	good	225	[28]	
Amundsen Bay	50.55	−66.78	20/10/2009	94	88	poor	250	[39]	Small, difficult to assess
Kloa Point	57.28	−66.64	13/11/2009	3521	3283	good	4500	[38]	
Fold Island	59.32	−67.32	14/10/2009	228	213	good	348	[38]	
Taylor Glacier	60.88	−67.45	21/10/2009	556	519	fair	2900	[11]	Some smearing over colony
Auster	63.98	−67.39	25/10/2009	8422	7855	poor	11000	[11]	
Cape Darnley	69.7	−67.88	15/10/2009	3713	3465	good	5000	[40]	
Amanda Bay	76.83	−69.27	13/10/2009	7315	6831	good	9000	[38]	
Haswell Island	93.01	−66.52	27/08/2009	3482	3247	poor	17000		Multispectral image bad, reanalysed with panchromatic image
Shackleton Ice Shelf	96.02	−64.86	10/10/2009	6937	6471	good	Uncounted		
Bowman Island	103.07	−65.16	26/10/2009	1724	1609	good	Uncounted		Good image
Peterson Bank	110.23	−65.92	24/11/2009	0	0	NA	1000	[18]	Late image, colony dispersed
Dibble Glacier	134.79	−66.01	12/10/2009	13377	12476	fair	Uncounted		Analysis of panchromatic only
Point Geologie	140.01	−66.67	01/10/2009	2632	2456	poor	2300	[11]	Streaking in panchromatic band
Mertz Glacier	146.62	−66.892	17/11/2009	5122	4781	poor	Uncounted		Huddles small and difficult to assess
Davis Bay	158.49	−69.35	11/10/2009	1870	1745	good	Uncounted		
Cape Washington	165.37	−74.64	16/10/2009	12663	11808	good	16822	[16]adults	Good image, lots of guano, may be underestimate
Beaufort Island	167.02	−76.93	12/10/2009	1758	1641	poor	1312	[16] adults	colony in shadow, difficult to differentiate
Franklin Island	168.43	−76.18	13/10/2009	8101	7561	good	2460	[16]adults	probable over-estimate
Cape Crozier	169.32	−77.46	11/10/2009	325	303	good	437	[16]adults	Small colony, image OK.
Coulman Island	∼169.61	∼−73.35	16/10/2009	27114	25298	fair	31432	[16]adults	Streaking in panchromatic band
Cape Roget	170.59	−71.99	16/10/2009	10186	9505	fair	7207	[3] chicks counted1996	Some streaking in panchromatic band; results may be overestimate
			**Total**	**238079**					

Table 1 presents the locations and best population estimate (BE) for each emperor penguin colony in the survey. The table also gives the image quality and the most recently published count for the colonies that have been previously counted with corresponding references.

Here we present the first synoptic survey of the entire population of a single species (breeding in a single year) using satellite remote sensing. Emperor penguins are particularly suitable for such a project because they breed at a relatively small number of sites and they breed mainly on sea ice where they have high contrast with their surrounding environment, making them easier to count in remote sensing imagery. Furthermore, our current knowledge of their global breeding population is limited. Finally, their predicted future decline due to climate change means that accurate current population assessments are needed to model their population dynamics.

Using Very High Resolution (VHR) satellite imagery we set out to:

1. Complete the survey initiated by the use of Landsat imagery [4] so that the entire Antarctic coastline has been surveyed by remote sensing for emperor penguin colonies.
2. Assess the population at every breeding emperor penguin colony.
3. Present a single breeding population figure from one synoptic count.

## Materials and Methods

### Data acquisition

To assess whether a penguin colony could be detected on an image and whether the image could be analysed, we examined un-georeferenced quick-looks from the QuickBird, WorldView-2 and Ikonos satellites. These quick-looks have a nominal resolution of ∼10 m, and therefore show greater detail than corresponding Landsat ETM images (see http://browse.digitalglobe.com/imagefinder/main.jsp for examples). Where evidence of emperor penguins was found, VHR satellites were tasked to collect images at these locations between September and December 2009, focussing on where colonies were previously thought to exist [11], [12]. The whole Antarctic coastline was assessed during the emperor penguin breeding season. Specific focus was given to sites where new colonies had been identified [15], [17], [18], [19], [20], and sites where there were unconfirmed sightings [11], [12], as well as locations where the previous Landsat survey had failed to acquire usable imagery of previously known sites [15].

Using this method, 51 possible sites were identified (46 from Table 1 and a number of other possible sites that eventually proved negative). Full resolution images for these sites were then uploaded and assessed to confirm whether an emperor penguin colony was present. All except one of these images were taken in the 2009 breeding season between late September and early December. The one exception was a newly found colony on the Rupert Coast (75.38°S latitude, 143.3°E Longitude), which was discovered too late in the season to acquire usable imagery. In this case imagery from the 2008 breeding season was used. Of the other 43 colony sites counted in this survey, 41 were assessed during a 54 day window between early October and late November (see Table 1). Thus, all known, or suspected breeding sites located on the fast-ice have now been examined for the presence of emperor penguin colonies.

### Analysis

**Figure 1:**
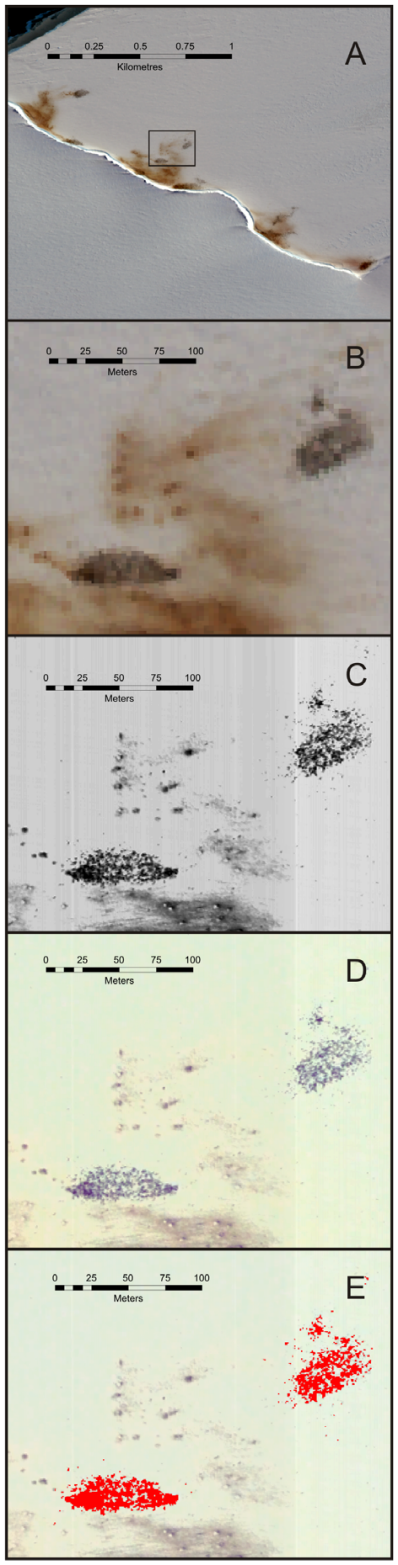
Example of imagery used in analysis. A: Multispectral QuickBird image of the emperor penguin colony at Windy Creek, Halley Bay, Antarctica. Black box indicates the area of images B–E below. B: Detail of multispectral image showing area of penguins as black/grey pixels and guano in brown. Although there is good differentiation between penguins and guano the coarse resolution of the multispectral image (2.54 m cell size) means that individual penguins cannot be identified and limits the usefulness of the image. C: Detail of the panchromatic band of the corresponding QuickBird image. The higher resolution (61 cm) gives better detail of the penguin area, but many of the penguin pixels have the same value as the areas of guano and therefore are difficult to separate using a classification index. D: Detail of the corresponding pansharpened QuickBird image. A histogram stretch has been used to maximize the difference between penguins and guano. Using this method the image retains the detail of the panchromatic image while keeping the colour differentiation of the multispectral image. E: Results from the supervised classification analysis of the pansharpened QuickBird image with the area classified as penguins shown in red.

QuickBird imagery has a resolution of 61 cm (at nadir) in the panchromatic band and 2.44 m resolution in the four multispectral bands (blue, green, red, and infrared). Emperor penguins show as single or multiple pixels in the panchromatic band. Where penguins are dispersed, individuals can be identified and counted. However, in the majority of cases penguins group into close clusters and their shadows overlap, meaning that individuals cannot be differentiated and a different approach is needed. Figure 1 shows an example of the high resolution imagery used in our analyses.

We used a multivariate supervised classification implemented in ArcGis™ v9.3 (ESRI®, 1999–2006) on QuickBird satellite images to assess the numbers of penguins at each colony. In previous work using this approach on the panchromatic band of VHR imagery, large errors were evident between estimated and actual counts [16], [21]. This was partially due to the problems of differentiating between penguins, shadows and guano (for an expanded discussion see Barber-Mayer et al [16]). For example, Barber-Mayer et al [16] encountered difficulties at the Cape Washington colony where the emperor penguins remained in large clusters in or around guano stained areas. Here an absolute deviation of 128% between the known and predicted count was found in 2005. This large deviation was attributed to the problem of differentiating guano from penguins in the panchromatic image bands. We have therefore modified the previous methods used by Barber-Mayer et al [16] by pansharpening the imagery (using an intensity/hue/saturation method). This results in a four band 61 cm resolution image that allows for much greater differentiation between guano, shadows and penguins. This process was carried out on the eight images from 2005 and 2006 that were used by Barber-Meyer et al [16]. These images were compared to aerial photographs taken simultaneously with the satellite imagery where adults were counted. A further three colonies (also adults only) were also counted. The three new counts were determined from vertical aerial (Smith Peninsula) or ground based photography (Amanda Bay and Fold Glacier) in the corresponding month as the satellite images from 2009.

Our processing routines may be summarised as follows. Each image was clipped to an area of interest and features within the image were classified into a number of classes. The number of classes depended upon each individual image and ranged between two and six, but most commonly four classes were used. The image classes used were: penguin, snow, shadow, guano, sometimes lighter snow and lighter penguins in areas of more contrast. In areas of different lighting conditions or where image banding (strips of different contrast on the image) occurred the colony was cropped into separate areas and multiple classifications conducted. The supervised classification process depends upon human interpretation to differentiate whether a pixel area is penguins or not. In some images, especially those with deep guano staining, this interpretation was more difficult and results will be less reliable in these areas (see Table 1 for details of each image). The method is iterative and usually several attempts were required before a good match between observed penguins and classified penguin area was obtained. When the area represented by penguins was determined, we converted the “penguin area raster” to a vector polygon within a GIS and reprojected the vector file to an equal-area projection. We then derived the true ground area represented by penguins at each colony by using the robust regression equation that was derived.

This approach (supervised classification) was then applied to all images of colonies obtained in 2009. The statistics from the robust regression were used to convert the area of penguins to population numbers for each site. The overall population figure includes counts from 16 previously uncounted colony sites.

### Statistical procedure

**Figure 2:**
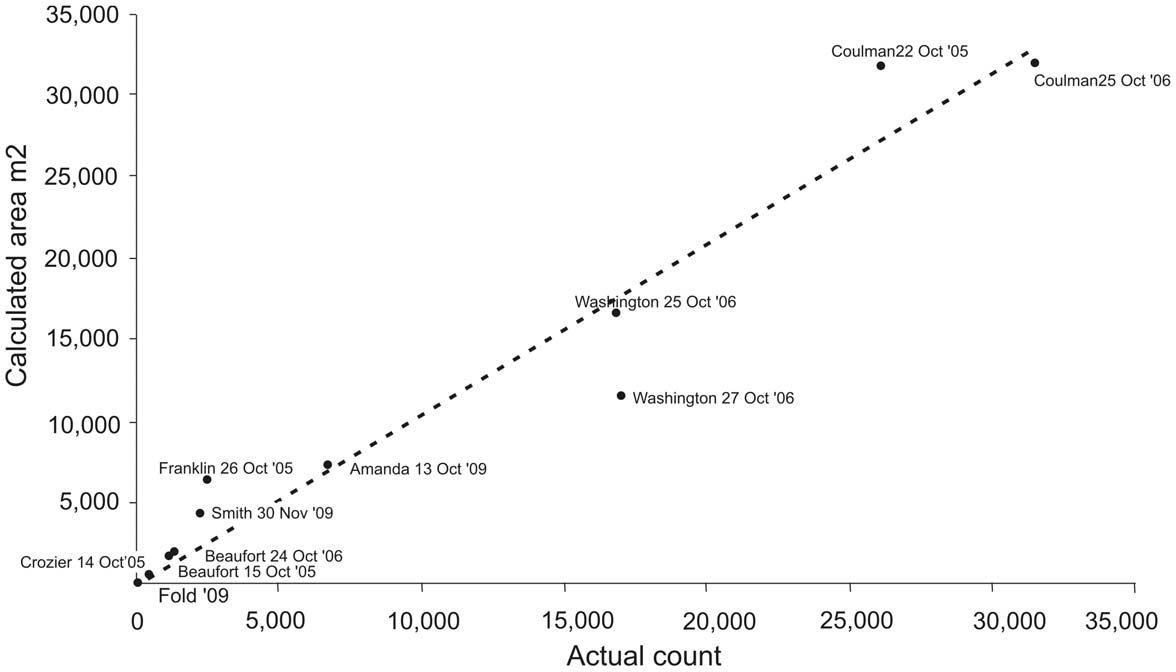
Regression plot based on the eleven ground truthing sites. The slope of the regression was 0.933 (SE = 0.046). Ground truth sites: Co6. Coulman Island 2006, Co5. Coulman Island 2005, Wa6. Cape Washington 2006, Wa5. Cape Washington 2005, Am. Amanda Bay 2009, Sm. Smith Peninsula 2009, Fr. Franklin Island 2005, Be6. Beaufort Island 2006, Be5. Beaufort Island 2005, Cr. Cape Crozier 2005.

The relationship between the colony area (total of all birds) and the number of adult birds present at a colony was estimated using robust linear regression (see Figure 2) with data from a sample of colonies for which both satellite area estimates were available and direct counts. Robust regression was used as this minimises the influence of outliers in the response variable, explanatory variable, or both. The model estimated a slope coefficient with SE (0.0464) but no intercept: this is in keeping with the truism that zero birds will occupy zero area. This was confirmed using a regression model excluding the intercept as this resulted in a negligible increase in variance. This model was fitted using the rlm function from the MASS library in R (R 2.8.0).

Population size estimates and confidence intervals around these were estimated for each colony using a Monte Carlo procedure. Simulated slope values were selected randomly from a normal distribution defined by a slope coefficient (0.933), the residual standard error (1851), residual degrees of freedom (10) and unscaled variance-covariance matrix of fixed effects (3.915766e-10) using the mvrnorm function in the R base package [22].

A slope value was generated for each colony and multiplied by its area to produce a population estimate for each, and these were summed to produce a global breeding population estimate. This was repeated 10,000 times, and the mean, 2.5 and 97.5 percentiles were calculated to represent the lower 95% and upper 95% confidence intervals (respectively) of the number of birds present at each colony, and globally.

## Results

We estimated a total population size of 238,079 adults present in all colonies in 2009, with 95% confidence intervals of 217,336 and 258,788 (see Table 1). We confirm the existence of 37 of the 38 colonies found in the previous Landsat study [15]. Our new survey also detected four new colonies (Brownson Islands, Dolleman Island, Dibble Glacier and Rupert Coast), and three previously suspected colonies [14] (Shackleton Ice Shelf, Bowman Island and Lazarev Ice Shelf). Two colonies remain uncounted; at Ledda Bay previous Landsat imagery from 1999 had identified a small colony, but in subsequent years early break up of fast-ice in the area has meant that no colony was present when there was coincident high resolution satellite imagery. The second location at Peterson Bank [18] was identified by air and ground survey in 1994. The corresponding QuickBird image in the 2009 breeding season was taken on 24 November and at this site the fast-ice had already retreated to the edge of the site and the majority of the colony had already departed. This colony probably still exists, but may have been unsuccessful in breeding in 2009. As earlier imagery of the area does not exist it is impossible to add an accurate estimate of numbers from this colony to our survey.

This makes a total of 46 colony locations around the coast of Antarctica. Note that the Dion Island colony is no longer believed to be occupied [8] and is not included (see figure S1 for distribution of population).

**Figure 3:**
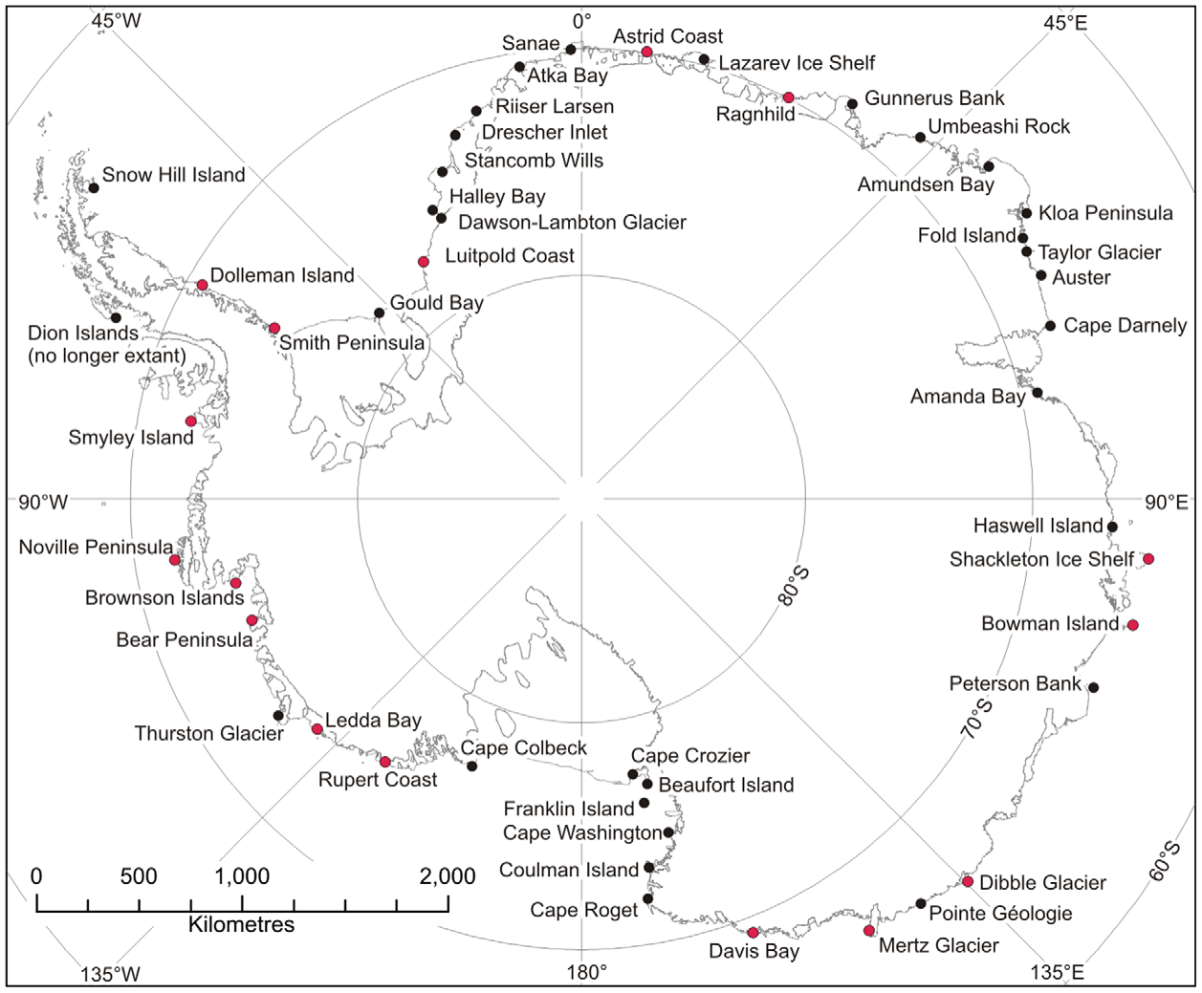
Distribution of emperor penguin colonies in Antarctica, see **Table 1** for details of each colony. Red dots refer to those colonies with no previous population estimates.

As previous population estimates did not take account of 16 of the 46 colonies (see Figure 3), and many previous counts were of poor quality and widely separated in time [11], [12], [23] these historical estimates cannot be considered representative of the total breeding population of emperor penguins (previous counts are given for comparison in Table 1). Our new global estimate may plausibly be used for calculation of future global population trends.

## Discussion

### Colony Detection

To determine whether any other unknown colonies have been missed is difficult; the variability in Antarctic sea-ice conditions means that in some locations sea-ice may have broken up early removing any evidence of a colony (as in the case of Ledda Bay). Also, image quality and cloud cover may make identification from ∼10 m imagery difficult. Finally, smaller colonies with less than 200 individuals may exist but these are more difficult to identify using imagery at this resolution. We believe that the number of small colonies will be limited as small groups are less likely to be able to huddle effectively during incubation [24]. Although a minimum effective huddle size has not yet been established, this limitation must exist, and penguins that cannot huddle effectively may suffer greater energy demands and thus greater weight loss and higher adult male mortality during the winter fast. The biological disadvantages of small colonies suggest that their number should be limited [32], [33], and although there may be a number of small colonies missing from this survey their contribution to the overall total population size is expected to be small. Any associated error on our overall population estimate should be minimal and probably within the confidence limits of our current global population estimate.

### Accuracy and uncertainty

Our results provide a new approach for assessing emperor penguin population numbers, though we believe some issues still need to be resolved. With future developments in ultra high resolution imagery, some of these issues will be naturally resolved. With existing capability, residual uncertainty derives from a number of sources, summarized in Table 2. These can be divided into (A) methodological error and (B) natural variability. Methodological errors can be divided into four types and are discussed below:

**Table 2:** Sources of error.

	Procedure	Result	Source of variability	Variability	Notes and suggestions for future work
1	Supervised classification	Area of penguins at each colony	Interpretation error: manual interpretation of which pixels constitute penguins as opposed to snow, guano or shadow. Variability here stems from being able to accurately determine penguins in the image, and repeatability between operators.	Less than 10% with most imagery but progressively worse with poorer imagery. Can be as much as 50% out in worst cases.	Depends upon the quality of the imagery. We suggest that future satellite acquisitions should avoid images with heavy guano staining.
2	Chick/adult area assumption	Area of adults at each colony	Chick adult ratio error: we make the assumption that the ratio of pixels showing as penguin in the satellite imagery remains constant to the number of adult pairs: i.e. That the area of larger chicks and fewer adults seen late in the season (November) is equal to the area of adults seen by the satellite earlier in the season (September, when chick are virtually invisible to the satellite).	Unknown at this stage, but the high correlation in good imagery from robust regression analysis confirms that the assumption is broadly true.	We suggest further work is needed to assess the variability. At present there is not enough ground truthing linked to satellite imagery over the period when the imagery is acquired.
3	Ground truthing estimates	Number of adults at selected colonies	From ground counts a mixture of error sources, mainly the error associated with counting an areas and scaling up to the whole colony. In aerial counts there can be variability in the manual interpretation of how many penguins (especially chicks) are on an image.	Approximate variability of ground truthing is around 10% using aerial photography, but can be higher for ground counts especially at larger colony sites	Low level vertical aerial photography is recommended to minimize ground truthing errors.
4	Statistical analysis	Estimated of adults at each colony at time of image	Statistical error: conversion of the pixels to penguins relies on a regression between area identified as penguin and the number of adults from ground truthing. Enough good ground truthing, concurrent with satellite imagery must be available to make this regression accurate.	1.75% based on Monte-Carlo analysis	More ground truthing over the entire season is recommended to improve the statistical procedure.
5	Seasonal assumption	Autumn population estimate	errors in the ground truthing and fluctuations between the dates of the ground truthing and satellite imagery	How this varies on a daily or weekly basis is at present unknown	Data from colonies where counts on seasonal variability would be useful. Especially if data exists on daily and weekly fluctuations in adult and chick numbers.
6	yearly population estimate	population estimate for 2009	Conversion between spring population and total population. Literature suggests that only 10% of birds are non- breeders	No variability estimate in literature	Further investigation required. Ground data from long term monitoring sites needed.
7	Inter-annual variability	Mean population estimate	Inter-annual changes at each emperor colony	Different estimates between colonies. Possibly size dependent (see text)	Monitor all colonies over multiple years by satellite to assess population change

The various sources of error; see section on Accuracy and uncertainty in the Discussion for further details of each area.

A.1. Supervised classification procedure: based upon the difficulty in differentiating penguins from guano or shadow, and from differing densities of penguins in clusters classed as penguin. This error source is compounded by manual interpretation inherent in the supervised classification procedure. To test the variability between operators when classifying pixels, four sites were classified by three different people. Results showed that the CV% around population estimates for individual colonies is low for colonies where there is good imagery (2.5 CV%), but becomes progressively worse with increasingly poor imagery. The quality of imagery is dependent upon contrast levels, whether the penguins are in shadow and if there is heavy guano staining. Errors in images with heavy guano staining such as from multispectral imagery at Haswell Island (original estimate of 50 CV%) can be large and almost certainly resulted in an over-estimate of penguin numbers at this site. Images such as this were the exception though; most colonies (24 out of 42 sites analysed) had very good imagery. (In the case of the Haswell Island image, the bad quality of the original multispectral image forced us to acquire an additional panchromatic image from earlier in the season in late August upon which our estimate for this colony is calculated).Based on a classification of each image by the user operator, image quality was classified into three quality groups, with each being assigned a corresponding level of variability; Table 3 shows the corresponding image classifications: good (2.5 CV%), reasonable (7.5 CV%) poor (15 CV%). To estimate the CV% of the total survey each pixel classed as penguin was attributed with a reliability estimate based upon these classes (see Table 3). The average CV% due to the image quality for all the pixels in the whole survey was calculated using this combined value, giving a value of 5.59CV%. Future surveys should attempt to acquire imagery with the minimum of guano staining to minimize operator error.

**Table 3:** Uncertainty estimates.

		Regression uncertainty				Image uncertainty		
Name	Area	BPE	UCI	LCI	%CV	Image Quality	%CV	Total CV
Cape Colbeck	12262	11438	10409	12442	8.89	good	2.5	306.6
Rupert Coast	1660	1550	1413	1685	8.78	good	2.5	41.5
Ledda Bay	0	0	0	0	8.62	NA	7.5	
Thurston Glacier	3205	2989	2725	3250	8.78	good	2.5	80.1
Bear Peninsula	10144	9457	8625	10270	8.7	good	2.5	253.6
Brownson Islands	6140	5732	5243	6226	8.58	poor	15	921.0
Noville Peninsula	3822	3568	3254	3876	8.71	poor	15	573.3
Smyley	6496	6061	5527	6604	8.88	good	2.5	162.4
Smith	4307	4018	3670	4366	8.66	good	2.5	107.7
Dolleman	1737	1620	1477	1764	8.87	good	2.5	43.4
Snowhill	2321	2164	1974	2351	8.7	poor	15	348.2
Gould	8833	8242	7519	8951	8.69	good	2.5	220.8
Luitpold	6969	6498	5944	7064	8.62	good	2.5	174.2
Dawson	2784	2597	2370	2828	8.81	good	2.5	69.6
Halley	24127	22510	20583	24444	8.58	good	2.5	603.2
Stancomb	5849	5455	4982	5922	8.61	fair	7.5	438.7
Drescher	2469	2305	2106	2502	8.6	fair	7.5	185.2
Riiser	4304	4013	3659	4372	8.88	fair	7.5	322.8
Atka	10355	9657	8807	10479	8.66	good	2.5	258.9
Sanae	3423	3193	2913	3469	8.71	good	2.5	85.6
Astrid	1467	1368	1249	1487	8.71	poor	15	220.1
Lazarev	881	821	748	892	8.74	fair	7.5	66.1
Ragnhild	7362	6870	6277	7461	8.62	good	2.5	184.1
Gunnerus	4989	4652	4237	5054	8.77	fair	7.5	374.2
Umbeashi	156	146	133	158	8.7	good	2.5	3.9
Amundsen Bay	94	88	80	95	8.67	poor	15	14.1
Kloa Point	3521	3283	2994	3565	8.7	good	2.5	88.0
Fold Island	228	213	194	232	8.87	good	2.5	5.7
Taylor Glacier	556	519	474	563	8.6	fair	7.5	41.7
Auster	8422	7855	7168	8556	8.83	poor	15	1263.3
Cape Darnley	3713	3465	3162	3766	8.72	good	2.5	92.8
Amanda Bay	7315	6831	6228	7425	8.76	good	2.5	182.9
Haswell Island	3482	3247	2958	3537	8.91	poor	15	522.3
Shackleton Ice Shelf	6937	6471	5918	7041	8.68	good	2.5	173.4
Bowman Island	1724	1609	1467	1748	8.74	good	2.5	43.1
Peterson Bank	0	0	0	0		NA		
Dibble Glacier	13377	12476	11376	13587	8.86	fair	7.5	1003.3
Point Geologie	2632	2456	2242	2670	8.7	poor	15	394.8
Mertz Glacier	5122	4781	4370	5208	8.77	poor	15	768.3
Davis Bay	1870	1745	1589	1895	8.79	good	2.5	46.8
Cape Washington	12663	11808	10790	12843	8.69	good	2.5	316.6
Beaufort Island	1758	1641	1497	1781	8.67	poor	15	263.7
Franklin Island	8101	7561	6900	8212	8.68	good	2.5	202.5
Cape Crozier	325	303	276	330	8.91	good	2.5	8.1
Coulman Island	27114	25298	23116	27486	8.64	fair	7.5	2033.6
Cape Roget	10186	9505	8694	10331	8.61	fair	7.5	764.0
	**255202**	**238079**	**217336**	**258788**	**1.75**		**5.59%**	**14273.8**

Table 3 gives details of the estimated statistical uncertainties associated with each colony. This is based on the robust regression analysis and the image quality of each VHR image. The uncertainty from the robust regression is estimated using Monte Carlo analysis (see Statistical Procedure section of the main text). The uncertainty based upon the image quality has been estimated using multiple analyses of images of differing quality. From this the survey has been broken into four classes as discussed in the Accuracy and uncertainty in the Discussion section.

A.2. Chick versus adult assumption: Most of our images (39 of 42 sites analysed) were taken over a 54 day window in the chick rearing season. At this time there is a mixture of adults and chicks at the site. Chick mortality during this period is low [3]. At the start of the period of our image acquisition there will be one adult per chick [31], at this time chicks are small or hidden and make up very little of the area classified as “penguin” in our supervised classification analysis. Later in the season chicks have emerged from under the feet of adults and are larger. At this stage they make up more of the pixels classified as “penguin” in our analysis. Conversely, the ratio of adults to chicks has diminished as more adults have left the colony to forage at sea. We make the assumption that in the 54 day window of image acquisition the ratio of pixels showing as penguin in the satellite imagery remains approximately constant to the number of adult pairs: i.e. That the area of larger chicks and fewer adults seen late in the season (November) is equal to the area with more adults seen by the satellite earlier in the season (October). This assumption needs to be tested, but at present not enough ground truthing concurrent with satellite imagery is available across the period to test how this affects the accuracy of our estimate.

A.3. Ground truthing estimates: Our regression analysis is based on the assumption of accurate ground truthing. In reality, ground truthing from ground counts or aerial photography also has inherent errors. Two sources of ground truthing have been used; aerial photography and ground counts. Estimations of variability in aerial photography counts indicate errors of +/−10%. This tends to be independent of colony size. With ground counts there is variability in both operator estimate and scaling errors.

A.4. Statistical analysis errors: conversion of the pixels to penguins relies on a regression between area identified as penguin and the number of adults from ground truthing. Enough good ground truthing, concurrent with satellite imagery must be available to make this regression accurate. The low Standard Error (0.0464) of the robust regression line in our study suggests that the relationship between area of penguins and the number of adults is consistent, and that other inherent errors (see 1 to 3 above) are small. Confidence in the levels of reliability is high for the population estimates for individual colonies, with confidence limits of ∼8.7%. Methodological errors will reduce in the future with the advent of even higher resolution imagery and additional ground truthing.

B. Natural variability: We make the assumption that at the time that the satellite imagery was taken, half of the adult breeding population would be present at the colony [31]; that is, our figure potentially represents the number of breeding pairs. Our initial estimate of 238,079 can therefore be considered to represent a count of breeding pairs that have successfully hatched a chick and raised it until at least October. Converting this figure to an overall population estimate brings further sources of variability. Uncertainty associated with naturally occurring fluctuations in penguin numbers stem from both seasonal and daily variation in the numbers of adults and chicks.

Our count only includes adult birds at the breeding site. Numbers of adults vary less on an inter-annual basis than that of chicks and are therefore a more accurate metric of population size [33]. Previously published work suggests that total chick mortality can be as high as 90%[25], [33] especially where storm events result in total breeding failure [26](total chick loss would result in the early dispersal of the colony, and this is a possible reason why the Peterson Bank colony did not exist at the time of imaging in late November). Our estimate does not include juveniles or non-breeding adults not present at the colony, or birds that have attempted to breed (present in May or June at the colony site) and have since departed. The percentage of birds remaining at the colony site after egg loss (egg loss is estimated to be approximately 20% of eggs laid with a SD of 6.4% [33])is low; typically less than 1%. Egg loss variability is one of several sources of potential error that must be included when converting a figure of adults at the colony site in October/November to a total population figure.

A better metric of population size would be a count of all colonies in June, when one male per breeding couple is at the colony site [33], all but five of our colony locations are south of the Antarctic circle and would be in 24 hour darkness at this time, so remote sensing with visible wavelengths of light at these colonies will be impossible. Even in the more northerly colonies at midwinter it is not feasible to use optical satellite imagery as the small time window, long shadows and low light levels result in a very limited number of very poor images, rendering accurate analysis impractical. The earliest possibility of gathering data from the most southerly emperor penguin colony (Gould Bay) is in late September or early October, so any continent wide survey that uses a consistent remote sensing methodology using visible wavelengths has to be after this date. Further ground truthing work to assess the number and variability of adults present in October/November compared to the actual breeding population present in June would aid our estimate.

### Numbers and interpretation

Mature emperor penguins breed almost every year [27]. The proportion of the breeding population each year has been estimated at 80% of the total population [26]. Using these estimates our October breeding population estimate therefore may represent a global population of around 595,000±81,753 individual birds, pre-breeding, i.e. before chicks of the year have hatched. The error figure is the sum of the regression error and potential variability associated with image quality, plus SD of egg loss variability, the variability of chick mortality between hatching and image acquisition is not included as this potential error source is presently unknown. However, it must be noted that our breeding estimate stems from for only one year (2009). Inter-annual population fluctuations at individual colonies can be as high as 30%, and changes of 10% or more per year are typical [3], [26], [28]. Recent population work gives the standard deviation of breeding adults at two well documented colonies; at Pointe Géologie over a fifty year period of CV 33.2%, and Haswell Island over a similar, but less well sampled, period as CV 22.4%. This magnitude of annual change should be identified by using the methods suggested in this paper and could be used in future to detect population trends. In the past, such variability was linked to a number of factors, which have been discussed in detail elsewhere [2], [8], [27], [29]. There is some indication that these factors are not independent, but act on the population as a whole [33]. Relationships with sea-ice variability, the Southern Annular Mode and prey and predator abundance all have the potential to modulate the annual breeding population. Therefore, to disentangle global, regional, or colony population trajectories associated with climate change from other influences will require long term ecological research. Such research is now becoming urgent as regional climate change is already impacting upon areas of West Antarctica and the Antarctic Peninsula [30] and colonies in this region may already be affected by the consequent loss of sea ice [8].

### Ecological implications

Current predictions [5], [6] suggest that trends in sea ice extent will alter in the second half of this century and that the annual average sea ice extent will diminish by 33%; most of this retreat is expected to occur in winter and spring [5], [6], with attendant risks for emperor penguins. Ainley et al [2] suggest that in the coming decades all colony sites located north of 70° South will become unviable for emperors. Ainley et al [2] equated this to approximately 40% of the world population. Our updated figures suggest that actually 34.8% of the total population breeds north of 70° South and is vulnerable to reductions in sea ice. However, an important consideration discussed in Trathan et al [8], is that warming is currently regional, and that a simple latitudinal gradient in the loss of sea ice is unlikely. Currently the loss of sea ice has been greatest from the West Antarctic Peninsula region. However, should the ozone hole indeed recover in the middle of this century, warming in East Antarctica is predicted to increase significantly [5], [6]. The ability to monitor populations using remotely-sensed data during consecutive breeding seasons and on a regional or global basis is a cost effective use of resources, particularly in comparison with aerial survey or ground counts. Such methods will therefore lead to a greater understanding of emperor penguins' current and future continued existence in areas affected by environmental change.

Understanding the causes of penguin decline will however require additional effort. Currently some of the important ecological factors needed to understand population change are not recorded on a regular or systematic basis. For example, fast ice provides a critical habitat for emperor penguins, yet this remains difficult to distinguish from pack ice at a regional and global scale. Developing new and appropriate remote sensing indices of pertinent environmental factors is therefore important, if we are to do more than simple measure population change.

### Expanding the methodology

Emperor penguins are suited to census by remote sensing for reasons mentioned above. Indeed, the results of this survey increase our knowledge of this species' population and distribution and provide a technique for long term monitoring. Though emperor penguins provide a particularly valuable model species, the techniques developed in this study may be applicable to a number of other animals. For example, some species of large herbivores with known migration patterns, especially those that are threatened by habitat degradation, climate change or human impact, may also benefit from the use of our methods. Many species are currently monitored by aerial survey, such methods are proportionally more expensive than satellite survey and have the potential to cause disturbance. The techniques used in this study, or similar techniques may therefore be appropriate for use with these species. The factors that make emperor penguins such a good model are useful criteria in assessing the suitability of other species for similar survey.

## Supporting Information

Figure S1 **Emperor penguin colonies 2009.** Size of circle relates to estimated number of pairs in each colony.
(EPS)
