# Correction: An Emperor Penguin Population Estimate: The First Global, Synoptic Survey of a Species from Space

**DOI:** 10.1371/annotation/32c246eb-3b73-4410-a44c-b41ddae11fc5

**Published:** 2012-04-26

**Authors:** Peter T. Fretwell, Michelle A. LaRue, Paul Morin, Gerald L. Kooyman, Barbara Wienecke, Norman Ratcliffe, Adrian J. Fox, Andrew H. Fleming, Claire Porter, Phil N. Trathan

There was an error in the Author Contributions designations. The correct Author Contributions are: Conceived and designed the experiments: PF PT GK CP AHF. Performed the experiments: PF ML CP. Analyzed the data: PF ML GK AJF NR. Contributed reagents/materials/analysis tools: PF ML PM CP NR. Wrote the paper: PF PT GK BW ML PM. Provided data: PM AHF GK BW. 

